# Obstructive Sleep Apnea Worsens Progression-Free and Overall Survival in Human Metastatic Colorectal Carcinoma

**DOI:** 10.1155/2021/5528303

**Published:** 2021-04-02

**Authors:** Donato Lacedonia, Matteo Landriscina, Giulia Scioscia, Pasquale Tondo, Incoronata Caccavo, Giuseppina Bruno, Guido Giordano, Annamaria Piscazzi, Maria Pia Foschino Barbaro

**Affiliations:** ^1^Institute of Respiratory Diseases, Department of Medical and Surgical Sciences, University of Foggia, Foggia, Italy; ^2^Medical Oncology Unit, Department of Medical and Surgical Sciences, University of Foggia, Foggia, Italy; ^3^Laboratory of Pre-Clinical and Translational Research, IRCCS, Referral Cancer Center of Basilicata, Rionero in Vulture, PZ, Italy

## Abstract

Sleep disorders have emerged as highly prevalent conditions, and along with improved understanding of such disorders, increased attention has gained the evidence that perturbation in sleep architecture and continuity may initiate, exacerbate, or modulate the phenotypic expression of multiple diseases including cancer. Furthermore, obstructive sleep apnea (OSA) has recently been implicated in increased incidence and more adverse prognosis of cancer in humans. This study was designed to confirm the high prevalence of OSA in human malignancies and assess its prognostic relevance in metastatic colorectal carcinomas (mCRCs). A prospective cohort of 52 subjects, affected by solid histologically confirmed metastatic malignancies, was analyzed, and among them, 29 mCRCs were studied for the prognostic role of OSA. OSA was diagnosed in 34.6% (18/52) of patients with a statistically significant difference in apnea-hyponea index between OSA and non-OSA subgroups (14.2 ± 12.2 vs. 2.1 ± 1.5, *p* < 0.01). Consistently, OSA was diagnosed in 34.5% (10/29) of mCRCs with lower rates of first-line therapy disease control in OSA compared to non-OSA patients (60% in OSA vs. 94.7% in non-OSA, *p*=0.03). Of note, progression-free and overall survival rates were significantly shorter in OSA (respectively, 9 and 22 months) compared non-OSA (20 and 40 months) mCRC patients (HR = 2.63; 95% CI 0.88–7.84, *p*=0.01 for PFS; HR = 3.93; 95% CI 1.13–13.73, *p* < 0.001 for OS). Finally, the multivariate analysis showed that OSA is an independent prognostic factor for PFS (*p*=0.0076) and OS (*p*=0.0017) in this cohort. Altogether, these data suggest that OSA is a potential clinical marker predictor of poor prognosis in patients with mCRC.

## 1. Introduction

Obstructive sleep apnea (OSA) is a disorder characterized by frequent subobstruction/obstruction of upper airways during sleep with intermittent hypoxia and sleep fragmentation. It is associated with systemic diseases such as hypertension, cardiac arrhythmias, cerebral and cardiovascular events, type 2 diabetes, postoperative complications, and several other morbidities [1–4]. It is well known that this association is due to systemic inflammation and sympathetic activation with production/secretion of a large spectrum of proinflammatory molecules. Recent studies in animal models and humans showed that sleep apnea and intermittent hypoxia may increase the risk of developing cancer [5] and may worsen its prognosis by increasing cancer progression and mortality [6]. Some studies established that there is a relationship between OSA and the risk of developing solid tumors [7, 8] and that patients with OSA usually have elevated cancer burden [9]. Furthermore, all-cause mortality risks are increased with sleep-related breathing **(**SDB) disorders severity, but the association between SDB and cancer mortality seems stronger and a link has been proposed between intermittent hypoxemia and the aggressiveness of human cutaneous malignant melanoma [7]. However, to date, the relationship between OSA and cancer prognosis is still debated [10].

Colorectal cancer (CRC) is a leading cause of cancer morbidity and mortality worldwide [11]. Unhealthy diet based on high fat intake, insufficient dietary fiber, high consumption of red or processed meat, and obesity represent common risk factors for CRC. Interestingly, many OSA patients share obesity, caused by unhealthy lifestyle, as common risk factor with CRC patients. Furthermore, much evidence suggests that OSA could increase the risk of developing CRC [12, 13], and a study cohort suggested that OSA may promote CRC development independently from obesity [14]. Thus, the present study was designed to confirm the frequency of OSA in human solid tumors and, more importantly, to establish a potential impact of OSA on prognosis of patients affected by metastatic CRC (mCRC).

## 2. Patients and Methods

This is a concept observational study in which a prospective cohort of 52 subjects, 27 males and 25 females, with a diagnosis of solid histologically confirmed metastatic malignancy, enrolled between October 2016 and April 2020 at the Medical Oncology Unit of the University Hospital of Foggia were studied for the presence of OSA. Patients' demographic characteristics are reported in Table 1. All subjects were followed for a median period of 32 months. In order to avoid possible interference between the neoplasm and the respiratory system, patients with chronic respiratory failure were excluded from the study. Subjects were consecutively recruited and underwent nocturnal cardiorespiratory monitoring at the time of diagnosis.

Main data collected were as follows: apnea-hyponea index (AHI), average SpO_2_, time with SaO_2_ lower than 90% (T90), and oxygen desaturation index (ODI). A questionnaire about comorbidities and daytime sleepiness (Epworth Sleepiness Scale, ESS) was administered. Data were analyzed by a physician expert in sleep medicine disorders who was not aware of the oncological condition of enrolled subjects. According to the presence of sleep apnea, the subjects were divided into 2 subgroups: OSA and non-OSA. Patients were considered positive for OSA in case of AHI >5. The prognostic relevance of OSA was studied in the subgroup of 29 mCRCs in order to establish a correlation between OSA and response to first-line therapy and cancer-specific survival. OSA was diagnosed before starting first-line chemotherapy (FOLFOX or FOLFIRI regimen combined with bevacizumab or anti-EGFR monoclonals selected based on RAS/BRAF mutational status). Demographic characteristics of mCRC patients are reported in Table 2.

The study was approved by the local Ethic Committee, and all participants signed a written informed consent. Fisher test or chi-square test was used to evaluate the differences between the two groups. Kaplan–Meier progression-free and overall survival curves (PFS and OS) were obtained. Response to therapy was evaluated according to RECIST criteria. Disease control rate (DCR) was defined as the sum of patients with stable or responding disease after first-line therapy. A multivariate logistic regression analysis was performed to identify factors influencing PFS and OS. The following factors were analyzed: presence/absence of OSA, age, sex, and cardiovascular and metabolic comorbidities, including diabetes, hypertension, ischemic heart disease, ictus cerebri, chronic obstructive pulmonary disease (COPD), and body mass index (BMI) (<25 vs. ≥25 kg/m^2^).

## 3. Results

OSA was diagnosed in 18/52 (34.6%) patients of the whole cohort (AHI 14.2 ± 12.2 vs. 2.1 ± 1.5, *p* < 0.01) and 10/29 (34.5%) patients of the mCRC cohort (Table 3). No significant differences were observed in OSA distribution according to age, sex, cardiovascular and metabolic comorbidities, body mass index (BMI) (<25 vs. ≥25 kg/m^2^), and tumor types (colorectal vs. noncolorectal).

The relationship between OSA and DCR, PFS after first-line therapy, and cancer-specific mortality was evaluated in the cohort of patients affected by mCRC (Table 2). Overall, 14 (48.2%) patients achieved a response to first-line therapy and 10 (34.5%) a disease stabilization, whereas 5 (17.3%) patients had a disease progression (Table 4). Interestingly, the response rate was constantly lower in OSA respect to non-OSA patients and presence of OSA significantly correlated with lower DCR (60% in OSA vs. 94.7% in non-OSA, *p*=0.03; Table 4). Median PFS and OS in the mCRC cohort were, respectively, 16 (95% CI 12–20) and 36 months (95% CI 28–43).

**Figure 1:**
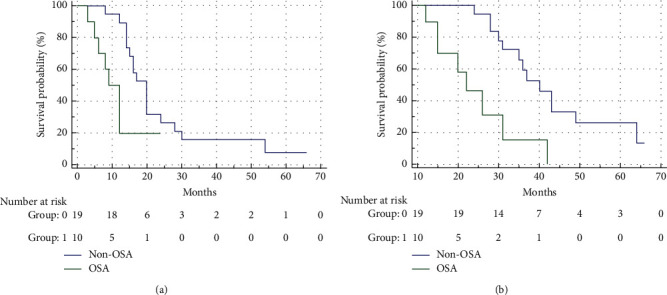
OSA impact on colorectal cancer clinical outcome. Kaplan–Meier progression-free (a) and overall (b) survival curves according to the presence/absence of OSA.

Interestingly, the PFS was significantly shorter in OSA (9 months) compared non-OSA (20 months) mCRC patients (HR = 2.63; 95% CI 0.88–7.84, *p*=0.01; Figure 1(a)). Consistently, OSA was significantly associated with reduced cancer-specific OS (median OS 22 months in OSA vs. 40 months in non-OSA, HR = 3.93; 95% CI 1.13–13.73, *p* < 0.001; Figure 1(b)). Finally, the multivariate analysis showed that OSA is an independent prognostic factor for PFS (*p*=0.0076) and OS (*p*=0.0017). Altogether, these results, although obtained in a small cohort of patients, suggest that OSA is a potential clinical marker predictor of poor prognosis in mCRCs.

## 4. Discussion

Much evidence suggests that sleep disorders are associated with increased risk of developing cancer and negatively impact on cancer prognosis [5, 6]. Based on this premise, this study enrolled a prospective cohort of patients with metastatic solid tumors, in majority of mCRCs, with the aim to confirm the high frequency of OSA among cancer patients and analyze the relevance of OSA in predicting cancer-specific prognosis in mCRCs. Our data suggest the following: (i) OSA is highly prevalent in cancer patients with no major difference between different cancer types, and (ii) OSA patients harboring mCRCs are characterized by lower rates of response to first-line therapy and shorter disease-free and overall survival compared to non-OSA patients. Noteworthy, the presence of OSA was shown to be an independent prognostic factor based on multivariate analysis.

These data raise several issues both at clinical and preclinical levels. Indeed, the association between OSA and cancer patients' outcome remains an open issue since there are many other confounding risk factors, which could affect prognosis [15]. Patients affected by OSA are often affected by obesity, which may contribute to chronic inflammation, altering the balance between oxidative stress, hormones, growth factors, and other mediators. Furthermore, several other confounding factors, such as cardiologic and metabolic comorbidities, besides cancer, may affect patient outcome [15]. In such a perspective, our data, obtained in a small, but homogeneous cohort of mCRCs, support the hypothesis that OSA may represent an independent predictor of poor response to standard first-line therapy and may negatively impact on cancer-specific survival. However, larger cohorts of patients are needed to establish a stronger relationship between OSA and specific CRC molecular characteristics/subtypes and between OSA and response to specific therapies (antiangiogenic versus other molecular targeted agents).

In a biological perspective, several mechanisms have been proposed to address the role of intermittent hypoxia in human carcinogenesis. It is established that cellular hypoxemia, usually present in both OSA and tumors, plays a central role in cancer progression by enhancing the expression of transcription factors, such as hypoxia-inducible factor (HIF-1), which activates genes involved in angiogenesis, oxidative stress response, genetic instability, invasion and metastasis, resistance to radiation, and chemotherapeutics [16]. In such a scenario, recent evidences, obtained in human CRC cell lines, suggested that HIF-1 is regulated at transcriptional level in response to intermittent hypoxia and not just by the posttranslational oxygen-dependent degradation pathway, as seen in chronic hypoxia [17]. Furthermore, other studies pointed out on the relationship between intermittent hypoxia and perturbation of tumor microenvironment. Indeed, hypoxemia influences the function of immune cells and macrophages present in tumor microenvironment, which, instead of attacking tumor cells, enhance the production of cytokines that promote tumor growth [18]. The contemporary production of reactive oxygen species generated during reoxygenation periods leads to activation of nuclear factor-kB (NF-kB) signaling that is associated with inhibition of apoptosis, synthesis/secretion of proliferation molecules, matrix metalloproteases, and angiogenetic molecules such as vascular endothelial growth factor (VEGF), which in turn enhance angiogenesis, tumor cell invasion, and metastatization [19]. The relevance of microenvironment in OSA-induced colorectal carcinogenesis has been recently pointed out by Gao et al., who suggested that intermittent hypoxia, chronic inflammation, and intestinal microbiota dysbiosis cooperate in CRC carcinogenesis in response to OSA [20]. Intermittent hypoxia influences also the expression of miRNAs, and some of them are known to be involved in cancer development or progression [21]. Finally, sleep fragmentation represents another factor influencing tumor initiation/progression since it causes epigenetic modifications of many circadian genes, which modify the expression of cancer-related susceptibility genes involved in cell division and DNA repair [22]. Altogether, these evidences are consistent with the general view that stress response is a driver of tumor progression [23] and that stress genes are prognostic factors in human malignancies [24]. In such a context, we previously reported that a stress-response gene signature based on the upregulation of TRAP1 gene network predicts the outcome of mCRCs [25].

## 5. Conclusions

This pilot study suggests that OSA may have a role in tumor progression and may predict poor response to therapy and clinical outcome in mCRC. Thus, it would be intriguing to speculate whether the dual targeting of cancer cells by anticancer agents in combination with continuous positive airway pressure CPAP, in patients with cancer and OSA, may provide a therapeutic perspective to improve response to therapy. In fact, we cannot rule out a beneficial effect of CPAP, based on its capacity to attenuate hypoxemia and modify the transcriptional profile of genes involved in cancer-related pathways [26]. In this perspective, further studies are needed to address the hypothesis that simultaneous targeting of intermittent hypoxemia and cancer cells may reduce cancer aggressiveness and improve the activity of anticancer pharmacological agents.

## Figures and Tables

**Table 1:** Demographic characteristics of patients.

Patients, *n*	52
Median age (range)	52.4 (43–87)
Females, *n* (%)	25 (48.1)
Males, *n* (%)	27 (51.9)

Tumor type, *n* (%)	
Colorectum	29 (55.9)
Breast	9 (17.3)
Lung	4 (7.7)
Ovary	4 (7.7)
Pancreas	2 (3.8)
Gallbladder	2 (3.8)
Prostate	1 (1.9)
Uterus	1 (1.9)

**Table 2:** Demographic characteristics of colorectal carcinoma patients.

Patients, *n*	29
Median age (range)	67 (44–87)
Males, *n* (%)	20 (69.0)
Females, *n* (%)	9 (31.0)

Metastatic pattern, *n* (%)	
Liver	20 (69.0)
Lung	11 (37.9)
Peritoneum	5 (17.2)
Bone	3 (10.3)
Others	2 (6.9)

First-line therapy, *n* (%)	
FOLFOX/bevacizumab	15 (51.7)
FOLFOX/anti-EGFR	10 (34.5)
FOLFIRI/bevacizumab	4 (13.8)

**Table 3:** OSA distribution according to severity and tumor type.

—	Non-OSA	OSA			
		All	Mild	Moderate	Severe
Patients, *n* (%)	34 (65.4)	18 (34)	10 (55.5)	3 (23.0)	5 (38.5)

Tumor type, *n* (%)					
Colorectum	19 (65.5)	10 (34)	7	2	1
Breast	5 (55.6)	4 (44.4)	2	1	1
Lung	0	4 (100.0)	3	—	1
Ovary	3 (75)	1 (25.0)	—	—	1
Pancreas	1 (50)	1 (50.0)	—	—	1
Gallbladder	2	1 (50.0)	—	—	—
Prostate	1	—	—	—	—
Uterus	1	—	—	—	—

**Table 4:** Response rate according to OSA distribution.

—	All, *n* (%)	OSA, *n* (%)	Non-OSA, *n* (%)
Complete response (CR)	1 (3.4)	0	1 (5.3)
Partial response (PR)	13 (44.8)	3 (30.0)	10 (52.6)
Stability (S)	10 (34.5)	3 (30.0)	7 (36.8)
Disease control rate (DCR)	24 (82.7)	6 (60.0)	18 (94.7)
Progression (P)	5 (17.3)	4 (40.0)	1 (5.3)

## Data Availability

The data used to support the findings of this study are included within the article.

## References

[1] Yaggi H. K., Concato J., Kernan W. N., Lichtman J. H., Brass L. M., Mohsenin V. (2005). Obstructive sleep apnea as a risk factor for stroke and death. *New England Journal of Medicine*.

[2] Marin J. M., Carrizo S. J., Vicente E., Agusti A. G. (2005). Long-term cardiovascular outcomes in men with obstructive sleep apnoea-hypopnoea with or without treatment with continuous positive airway pressure: an observational study. *The Lancet*.

[3] Babu A. R., Herdegen J., Fogelfeld L., Shott S., Mazzone T. (2005). Type 2 diabetes, glycemic control, and continuous positive airway pressure in obstructive sleep apnea. *Archives of Internal Medicine*.

[4] Lacedonia D., Carpagnano G. E., Patricelli G. (2018). Prevalence of comorbidities in patients with obstructive sleep apnea syndrome, overlap syndrome and obesity hypoventilation syndrome. *The Clinical Respiratory Journal*.

[5] Almendros I., Montserrat J. M., Ramirez J. (2012). Intermittent hypoxia enhances cancer progression in a mouse model of sleep apnoea. *European Respiratory Journal*.

[6] Abrams B. (2007). Cancer and sleep apnea-the hypoxia connection. *Medical Hypotheses*.

[7] Martinez-Garcia M.-A., Martorell-Calatayud A., Nagore E. (2014). Association between sleep disordered breathing and aggressiveness markers of malignant cutaneous melanoma. *European Respiratory Journal*.

[8] Campos-Rodriguez F., Martinez-Garcia M. A., Martinez M. (2013). Association between obstructive sleep apnea and cancer incidence in a large multicenter Spanish cohort. *American Journal of Respiratory and Critical Care Medicine*.

[9] Sillah A., Watson N. F., Schwartz S. M., Gozal D., Phipps A. I. (2018). Sleep apnea and subsequent cancer incidence. *Cancer Causes & Control*.

[10] Palamaner Subash Shantha G., Kumar A. A., Cheskin L. J., Pancholy S. B. (2015). Association between sleep-disordered breathing, obstructive sleep apnea, and cancer incidence: a systematic review and meta-analysis. *Sleep Medicine*.

[11] Bray F., Ferlay J., Soerjomataram I., Siegel R. L., Torre L. A., Jemal A. (2018). Global cancer statistics 2018: GLOBOCAN estimates of incidence and mortality worldwide for 36 cancers in 185 countries. *CA: A Cancer Journal for Clinicians*.

[12] Brenner R., Kivity S., Peker M. (2018). Increased risk for cancer in young patients with severe obstructive sleep apnea. *Respiration*.

[13] Zhang X., Giovannucci E. L., Wu K. (2013). Associations of self-reported sleep duration and snoring with colorectal cancer risk in men and women. *Sleep*.

[14] Lee S., Kim B. G., Kim J. W. (2017). Obstructive sleep apnea is associated with an increased risk of colorectal neoplasia. *Gastrointestinal Endoscopy*.

[15] Gozal D., Almendros I., Phipps A. I., Campos-Rodriguez F., Martínez-García M. A., Farré R. (2020). Sleep apnoea adverse effects on cancer: true, false, or too many confounders?. *International Journal of Molecular Sciences*.

[16] Carmeliet P., Dor Y., Herbert J.-M. (1998). Role of HIF-1*α* in hypoxia-mediated apoptosis, cell proliferation and tumour angiogenesis. *Nature*.

[17] Martinez C.-A., Kerr B., Jin C., Cistulli P., Cook K. (2019). Obstructive sleep apnea activates HIF-1 in a hypoxia dose-dependent manner in HCT116 colorectal carcinoma cells. *International Journal of Molecular Sciences*.

[18] Sato T., Takeda H., Otake S. (2010). Increased plasma levels of 8-hydroxydeoxyguanosine are associated with development of colorectal tumors. *Journal of Clinical Biochemistry and Nutrition*.

[19] Tammali R., Saxena A., Srivastava S. K., Ramana K. V. (2019). Expression of concern: aldose reductase inhibition prevents hypoxia-induced increase in hypoxia-inducible factor-1*α* (HIF-1*α*) and vascular endothelial growth factor (VEGF) by regulating 26 S proteasome-mediated protein degradation in human colon cancer cells. *Journal of Biological Chemistry*.

[20] Gao J., Cao H., Zhang Q., Wang B. (2020). The effect of intermittent hypoxia and fecal microbiota of OSAS on genes associated with colorectal cancer. *Sleep and Breathing*.

[21] Lacedonia D., Scioscia G., Pia Palladino G. (2018). MicroRNA expression profile during different conditions of hypoxia. *Oncotarget*.

[22] Haus E. L., Smolensky M. H. (2013). Shift work and cancer risk: potential mechanistic roles of circadian disruption, light at night, and sleep deprivation. *Sleep Medicine Reviews*.

[23] Landriscina M., Maddalena F., Laudiero G., Esposito F. (2009). Adaptation to oxidative stress, chemoresistance, and cell survival. *Antioxidants & Redox Signaling*.

[24] Rotblat B., Grunewald T. G. P., Leprivier G., Melino G., Knight R. A. (2013). Anti-oxidative stress response genes: bioinformatic analysis of their expression and relevance in multiple cancers. *Oncotarget*.

[25] Maddalena F., Simeon V., Vita G. (2017). TRAP1 protein signature predicts outcome in human metastatic colorectal carcinoma. *Oncotarget*.

[26] Chen Y. C., Chen K. D., Su M. C. (2017). Genome-wide gene expression array identifies novel genes related to disease severity and excessive daytime sleepiness in patients with obstructive sleep apnea. *PLoS One*.

